# Preliminary Findings of a Technology-Delivered Sexual Health Promotion Program for Black Men Who Have Sex With Men: Quasi-Experimental Outcome Study

**DOI:** 10.2196/publichealth.7933

**Published:** 2017-10-24

**Authors:** Charles H Klein, Tamara Kuhn, Danielle Huxley, Jamie Kennel, Elizabeth Withers, Carmela G Lomonaco

**Affiliations:** ^1^ Department of Anthropology Portland State University Portland, OR United States; ^2^ dfusion Oakland, CA United States; ^3^ ETR Scotts Valley, CA United States; ^4^ Department of Emergency Medical Services Oregon Health & Sciences University Portland, OR United States; ^5^ Department of Emergency Medical Services Oregon Institute of Technology Klamath Falls, OR United States; ^6^ Department of Sociology Portland State University Portland, OR United States; ^7^ University of California, San Francisco San Francisco, CA United States

**Keywords:** HIV, health promotion, sexuality, harm reduction, African Americans, sexual minorities, telemedicine

## Abstract

**Background:**

Human immunodeficiency virus (HIV) disproportionately affects black men who have sex with men (MSM), yet there are few evidence-based interventions specifically designed for black MSM communities. In response, the authors created Real Talk, a technology-delivered, sexual health program for black MSM.

**Objective:**

The objective of our study was to determine whether Real Talk positively affected risk reduction intentions, disclosure practices, condom use, and overall risk reduction sexual practices.

**Methods:**

The study used a quasi-experimental, 2-arm methodology. During the first session, participants completed a baseline assessment, used Real Talk (intervention condition) or reviewed 4 sexual health brochures (the standard of care control condition), and completed a 10-minute user-satisfaction survey. Six months later, participants from both conditions returned to complete the follow-up assessment.

**Results:**

A total of 226 participants were enrolled in the study, and 144 completed the 6-month follow-up. Real Talk participants were more likely to disagree that they had intended in the last 6 months to bottom without a condom with a partner of unknown status (mean difference=−0.608, *P*=.02), have anal sex without a condom with a positive man who was on HIV medications (mean difference=−0.471, *P*=.055), have their partner pull out when bottoming with a partner of unknown HIV status (mean difference=−0.651, *P*=.03), and pull out when topping a partner of unknown status (mean difference=−0.644, *P*=.03). Real Talk participants were also significantly more likely to disagree with the statement “I will sometimes lie about my HIV status with people I am going to have sex with” (mean difference=−0.411, *P*=.04). In terms of attitudes toward HIV prevention, men in the control group were significantly more likely to agree that they had less concern about becoming HIV positive because of the availability of antiretroviral medications (mean difference=0.778, *P*=.03) and pre-exposure prophylaxis (PReP) (mean difference=0.658, *P*=.05). There were, however, no significant differences between Real Talk and control participants regarding actual condom use or other risk reduction strategies.

**Conclusions:**

Our findings suggest that Real Talk supports engagement on HIV prevention issues. The lack of behavior findings may relate to insufficient study power or the fact that a 2-hour, standalone intervention may be insufficient to motivate behavioral change. In conclusion, we argue that Real Talk’s modular format facilitates its utilization within a broader array of prevention activities and may contribute to higher PReP utilization in black MSM communities.

## Introduction

### Background

Human immunodeficiency virus (HIV) has disproportionately affected black men who have sex with men (MSM) since the beginning of the epidemic. Today, nearly 40% of individuals living with HIV in the United States are African American, even though African Americans represent only 12% of the US population [1]. Among the more than 350,000 black men living with HIV, approximately half (53%) are MSM [2]. Since 2014, black MSM have been the subpopulation with the highest number of new HIV diagnoses [3], and a recent meta-analysis estimated black MSM incidence rates at 4.16% [4]. If these trends continue, 50% to 60% of black gay and bisexual men will receive an HIV diagnosis in their lifetime, nearly 3 times the percentage of gay and bisexual men overall [3,4].

Despite the devastating impact of HIV on black MSM, there are relatively few evidence-based HIV prevention interventions designed specifically for black MSM [5]. Nor are there any demonstrated efficacious technology-delivered interventions targeting black MSM, despite the growth of effective eHealth and mHealth sexual health programs in recent years [6]. Because funders such as Centers for Disease Control and Prevention (CDC) often require practitioners to use programs with demonstrated efficacy, the lack of evidence-based programs designed specifically for black MSM may limit our ability to respond to black MSM’s diverse sexual health needs, identities, and intervention format preferences.

### The Real Talk Program

Seeking to offer additional evidence-based HIV prevention options for black MSM and the providers who serve them, the investigators developed Real Talk in both face-to-face and computer or tablet-delivered formats, the latter of which is the focus of this study (see [7] for a detailed presentation of Real Talk’s product development and intervention content). Real Talk is loosely based on a popular suite of Afrocentric, group-level HIV prevention interventions developed for adult, teenage, and HIV-positive African-American women—SISTA, SiHLE, and WiLLOW [8]. The trilogy focuses on risk reduction strategies, skills building and peer support using a social cognitive theoretical framework within the context of the intersectionalities experienced by black women. These interventions are part of CDC’s Diffusion of Effective Behavioral Interventions (DEBI) library [9] and are also available in 2-hour long computer-delivered versions, all of which demonstrated preliminary efficacy in reducing HIV-related risks [10-12]. Given that black MSM face many of the intersectionalities addressed in the trilogy, including gender/sexuality power dynamics and racism in interactions with the mainstream (white) gay community, the developers believed that the trilogy’s Afro-centric empowerment approach might resonate with black MSM and similarly generate improved sexual health outcomes.

In adapting the SISTA/SiHLE/WiLLOW trilogy for black MSM, Real Talk positions HIV prevention within a growing gay health movement that defines sexual health as more than safer sex practices or the absence of disease [13-15]. Real Talk does this through affirming black MSM’s resilience in the face of intersecting forms of discrimination and oppression while acknowledging the reality of high levels of HIV prevalence among black MSM [16]. And because individuals vary in their ability—and indeed, desire—to engage in consistent condom use, Real Talk uses a sexual harm reduction framework that recognizes the many HIV prevention strategies that MSM use today. These include serosorting, negotiated safety agreements, and for HIV-negative men, pre-exposure prophylaxis (PReP) [17-19]. Our starting point is to meet people where they are [20] and not judge men because of their (unsafe) sexual behaviors. In addition, unlike many DEBI programs, Real Talk does not attempt to persuade participants to adopt a particular HIV risk reduction strategy or set of strategies (eg, 100% condom use with all partners, monogamous relationships, and decreasing the number of sexual partners). The program instead offers men a 6-step harm reduction tool to help them make sexual health decisions that are in line with their values, life objectives, and the current HIV prevention landscape outlined above. In this framework, condom use is an important, but not the only, sexual health promotion strategy. By being upfront about these realities, we believe Real Talk can more credibly engage men who might otherwise be less responsive to condom-focused interventions while nonetheless not discounting the importance of condoms as an HIV prevention strategy.

We created both the computer and face-to-face versions of Real Talk using an iterative agile product development process that included the following: (1) a Web-based needs assessment of national practitioners recruited through the National Minority AIDS Council in 2012, focus groups with black MSM in Atlanta and San Francisco in 2012-2013, and interviews with HIV prevention providers in these same cities in 2012-2013, (2) prototype testing of 2 activity components of the computer/tablet-delivered program with black MSM in 2013, (3) a run-through of the complete 12-hour face-to-face program in early 2014, and (4) input on design, activity format, and storyboards from a community panel of 6 black MSM throughout programming of the computer/tablet-delivered version in the second half of 2014. The final technology-delivered version of Real Talk plays on PC and Mac computers and Android mobile tablets, with WiFi necessary for optimal user experience. The program’s 6 modules take users approximately 2 hours to complete, in comparison to 12 hours for the face-to-face format (Multimedia Appendix 1). Users may stop at any point, resume where they left off, and if they desire, repeat the already completed activities. All modules combine audio narration, visual presentations, interactive components (eg, drop and drag, list creation, and scroll over pop-ups), games, and video clips of black MSM talking about their lives (Multimedia Appendices 2 and ). We have also consciously inserted humor within each module to maintain viewer interest (eg, the “mad scientist” presenter for Module 4’s Condom Laboratory) and to defuse potentially emotionally challenging topics (eg, Module 5’s communication style videos, which mirror the over-the-top role-plays that participants created in the 2014 face-to-face curriculum pilot tests).

Real Talk’s content builds on the three central themes identified in our formative research: (1) stigma, discrimination, and intersectionalities in the lives of black MSM, (2) the need for safe spaces and community, and (3) the need for sexual harm reduction approaches in HIV prevention programming [7]. For example, the opening “My Community” module situates sexual health within the broader context of men’s lives and includes self-reflections on user’s relationships with black, gay, and black gay communities and video content addressing racism in gay communities and homophobia within the family. These activities enable men to examine the tensions that can arise from balancing racial, sexual, and other identities and how these processes may affect sexual health decision-making. These themes are revisited in Module 3, where men reflect on stress and coping strategies; in Module 4, where men explore different harm reduction strategies in the unique contexts of their own lives; and in Module 7, where men examine what kind of relationships they would like to have and where they might turn to for sexual health and relationship support.

### Study Aims

This study aimed to determine whether a culturally tailored, computer/tablet-delivered, sexual health program can engage black MSM on sexual health issues, promote HIV risk reduction practices, and produce improvements in psychosocial factors linked to sexual risk behaviors. We hypothesized that, relative to the control condition, men in the Real Talk condition at follow-up would report the following: (1) higher levels of intention to reduce HIV risk, (2) increased HIV disclosure with partners, (3) higher levels of condom use for insertive and receptive anal sex, and (4) less risky sexual practices overall. These findings would provide preliminary support for the efficacy of Real Talk and offer organizations a technologically contemporary and easily scalable evidence-based HIV program for black gay men/MSM.

## Methods

### Recruitment

From June 2015 to May 2016, we conducted a quasi-experimental, 2-arm outcome study at 4 sites to test the preliminary efficacy of the computer/tablet-delivered version of Real Talk in reducing sexual health risks and improving psychosocial factors associated with sexual health (Portland State University IRB Protocol #153352). Two study sites were located in Florida, and one each in Georgia and New Jersey, and all sites have long histories of providing prevention and care services to black MSM. Due to the endogamous structures of black MSM communities [21] and the possibility of intervention effect contamination, we assigned sites to either the Real Talk condition (the 2 Florida sites) or the control condition (the Georgia and New Jersey sites). Our target enrollment was 276 men. Accounting for 15% study attrition, we projected a sample size of 240 men who would complete the baseline, condition, and 6-month follow-up. Based on our formative research and the epidemiological literature on black MSM sexual practices, we estimated that the control condition would report an average of 70% condom protected sex at the 6-month follow-up. Using alpha=.05 and a 2-sided test, this sample size would support the detection of a 10% difference in condom-protected sex between the control and intervention conditions with 80% power.

Sites recruited men through their existing client base, venue-based outreach, social media spaces, and snowball sampling. To be eligible, men were required to self-identity as black/African-American, be between the ages of 18 and 49 years, and report having had sex with a man in the past 3 months. We decided on a cutoff age of 49 years because of the following reasons: (1) the intervention does not include any specific content on aging and sexual health issues, (2) program aesthetics and role-play scenarios were directed toward the 20s to 40s age range, and (3) individuals in the 18 to 49 age range are on average more likely to be sexually active than their older counterparts. During the first session, participants completed a baseline assessment using a computer or tablet administered SurveyMonkey instrument, used Real Talk (intervention condition) or reviewed 4 sexual health brochures (the standard of care control condition), and completed a 10-minute user-satisfaction survey on their impressions of their respective study condition. Six months later, participants from both conditions returned to complete the follow-up assessment. The baseline and follow-up assessments were identical and assessed demographic characteristics; mental health and social support; HIV/STI (sexually transmitted infection) knowledge and prevention atittudes; partner communication; HIV/STI history; race, identities, and sexuality (intersectionalities); alcohol and drug use; and sexual behavior/risk reduction strategies.

Over the period of June to October 2015, 226 participants were enrolled in the study and completed the baseline assessment and their particular condition, with 106 men in the Real Talk arm and 120 men in the control (Multimedia Appendix 4). Participants received US $50 for completing the first session and US $75 for completing the follow-up assessment. A total of 140 participants returned for the 6-month follow-up assessment, 72 in the intervention condition (67.9% retention rate) and 68 in the control condition (56.7% retention rate). There were no significant sociodemographic differences between the 140 participants retained in the study at follow-up compared with the 86 men unavailable for the follow-up assessment. Men who completed the 6-month post assessment were significantly less likely, however, to report being connected to their families (94/140, 67.1% vs 186/226, 82.3%, *P*=.03) and having healthy relationships with their partners (90/140, 64.3% vs 199/226, 88.1%, *P*=.01) than the baseline sample. One hypothesis to explain this difference may be that socially isolated men may interact more frequently with community-based organizations to meet their psychosocial needs than their more social integrated peers and, as a result, be less likely to be lost to follow-up.

### Measures

#### Risk Reduction Intentions and Disclosure Practices

Risk reduction intentions over the past 6 months were assessed using 22, 1-5 scale, Likert items addressing risk reduction strategies (eg, condom use, serosorting, strategic positioning, pulling out, and not using condoms when positive partner is on ART or has an undetectable viral low) [22]. A 5-item index (alpha=.928) assessed men’s confidence in negotiating safe sexual practices with their partners, with higher scores indicating greater partner communication efficacy [23]. Twelve Likert-type items addressed disclosure-related issues (eg, “I will wait for my partner to tell me his status before I tell him mine,” “I will tell my partners my HIV status before I have sex with them,” and “I will sometimes lie about my HIV status to people I am going to have sex with”). Fifteen questions addressed intentions on sexual practices and risk reduction strategies for the next 6 months [22].

#### Condom Use and Other Risk Reduction Practices

Condom use and other risk reduction practices were assessed through questions addressing the following: (1) the number of times participants had engaged in particular risk reduction strategies for insertive and receptive anal sex, with separate sections for positive, negative, and unknown status male partners (8 questions per partner HIV status type) and (2) a 24-question examination on each of the participants’ last 3 sexual partners, including partner characteristics, discussion of HIV status, risk reduction practices for insertive and receptive anal sex, and reasons for not using condoms for topping and bottoming if applicable [22].

#### Sexually Transmitted Infections and HIV Status

Men’s HIV/STI history was assessed using self-reports of gonorrhea, chlamydia, syphilis, and human papillomavirus diagnoses in the past 6 months, date of last HIV test, result of last HIV test, PrEP use in self-reported HIV-negative men, and ART and viral load testing among self-reported HIV-positive men.

#### Psychosocial Mediators

We derived psychosocial mediators from the intervention’s underlying social cognitive and gender and power theoretical framework and the literature discussed in the introduction section of this study, with the goal of capturing potential changes in mental health, social support, and other factors that may mediate an individual’s HIV risk and risk reduction practices. All constructs were assessed using scales with satisfactory psychometric properties developed in evaluations of the face-to-face and computer-delivered versions of the SiSTA/SiHLE/WiLLOW trilogy [11,12,23,24] and behavioral studies with black MSM [18,22,25].

##### Mental Health and Social Support

Self-esteem was assessed using the 10-item Rosenberg Self-Esteem Scale (alpha=.885) [26] and the 18-item Ways of Coping Questionnaire [27]. We also created 14 yes/no questions addressing men’s social support from family, friends, work, and black and gay communities (5 questions on practical support, 5 on connectedness, and 4 on relationship quality).

##### Prevention Knowledge and Attitudes

Six true/false questions addressed HIV transmission risk knowledge (eg, “HIV is only transmitted through anal sex,” “STIs put people at a greater risk of HIV infection,” and “sheepskin condoms are better than latex condoms for preventing HIV transmission”). A 5-item index assessed negative attitudes to condom use (alpha=.585) [22], and several sections from the ASSORT! study instrument addressed attitudes toward HIV prevention [22]: importance of HIV risk reduction given the realities of effective HIV treatment (4 questions), relative responsibility of HIV-negative and HIV-positive men for HIV prevention (8 questions), and perceived HIV risk based on the race/ethnicity and age of partners (9 questions).

##### Identities and Intersectionalities

Fourteen yes/no questions compared the experiences of black men to gay men, women, and heterosexual men of different race/ethnicities. Two open-ended questions and 3 ranking questions examined experiences of discrimination. Nine questions asked where respondents socialize and meet partners, 4 questions measured the number of people who know they have sex with men (ie, friends, family, work, and overall), and the 45-item Aspects of Identity Questionnaire IV (alpha=.971) [28] assessed personal, relational, social, and collective identity orientations.

##### Alcohol and Substance Use

Two questions addressed alcohol consumption frequency (days used) and intensity (number of drinks per day) in the past 30 days. Four questions addressed substance use just before or during sex in the past 30 days (alcohol, poppers, downers, and painkillers), and 10 questions covered non-prescription substance use in the past 30 days for marijuana, hallucinogens, ecstasy, ketamine, GHB, methamphetamine, crack, powder cocaine, heroin, and erectile dysfunction medications.

#### User Satisfaction

Participants completed a 22-item user satisfaction survey immediately after viewing Real Talk (intervention) or reviewing the sexual health brochures (control). The user satisfaction included Likert-like scale questions on experience with the program or brochures (ie, enjoyment, presentation, held attention, and clarity) and intervention/material quality (ie, overall design, ease of use, usefulness of information, and potential to help people lower their sexual health risks). Open-ended questions addressed overall impressions, likes and dislikes, new information learned, and suggestions for improving Real Talk or the sexual health brochures.

### Data Analysis

Statistical analyses occurred in 3 phases. We first calculated descriptive statistics for sociodemographic variables, hypothesized mediators, and sexual behaviors. We then conducted bivariate analyses to assess differences between conditions, using *t* tests for continuous variables and chi-square test for dichotomous variables. In our third analytic stage, we constructed linear, negative binomial, and linear regressions to assess Real Talk intervention effects at the 6-month follow-up. Variables for which differences at baseline between study conditions were statistically significant (*P*<.05) and which were hypothesized to be linked to outcomes were included as covariates in the models. For continuous outcomes (eg, all scales and the Likert-scale risk reduction practice questions), we constructed separate linear multiple regression models and calculated mean differences, percent relative change, and the corresponding 95% CI and *P* values. For count variables (eg, # of times the participant used particular risk reduction strategies for HIV-positive, HIV-negative and unknown status partners), we constructed separate negative binomial regression models and calculated adjusted means, likelihood ratios, and the corresponding 95% CI and *P* values. For dichotomous outcomes (eg, used condoms during the last anal sex, all yes/no disclosure, social support, connectedness, and knowledge questions), we constructed multiple logistic regression models and calculated adjusted odds ratios, 95% CIs, and corresponding *P* values. Analyses were conducted using SPSS statistics version 23 (IBM Analytics, Armonk, New York).

## Results

### Participants

A total of 140 participants completed the baseline assessment, study condition, and 6-month post assessment. At baseline, participants had a mean age of 33 years. A total of 66 men (47%) reported being HIV-positive and 57 (41%) being HIV-negative, with the remainder not reporting their HIV status. Sixty-two men (44%) described their sexual identity as gay, 40 (29%) homosexual, 9 (6%) same-gender loving, 15 (11%) bisexual, and 4 (3%) heterosexual. For the sample as a whole, 65% of people in the men’s lives knew that they have sex with other men. In terms of the highest level of education, 12 men (9%) reported having less than a high school degree, 46 (33%) a high school diploma, 42 (30%) some college degree, 18 (13%) a 2-year or technical degree, 17 (12%) a 4-year degree, and 4 (3%) having completed graduate work beyond a 4-year degree. Participants’ incomes were below national averages, with 34 men (24%) reporting less than $6000/year, 24 (17%) between $6000 and $12,000/year, 33 (24%) between $12,000 and 24,000/year, 33 (24%) between $24,000 and 48,000/year, and 12 (9)% earning over $48,000/year.

Participants reported relatively low levels of stress (mean=2.4 on a 1 to 5 scale, with 5=a great deal of stress, SD=0.84251), high levels of self-esteem (mean=4.2 on a 1 to 5 scale, with 5=highest self-esteem, SD=0.80244), moderately high levels of self-efficacy (mean=3.9 on a 1 to 5 scale, with 5=highest self-efficacy, SD=0.76667), and moderate levels of coping skills (mean=3.26 on a 1 to 5 scale, with 5=the highest level of coping, SD=0.55940). A total of 94 men (67%) also said they had a healthy relationship with their families. Only 51 (36%) men felt that the black community accepts black gay men, whereas 85 men (61%) thought that the white gay community accepts black gay men. In terms of where men socialize, 105 (75%) men reported hanging out and meeting men online, 94 (67%) at gay bars, 92 (66%) at dance clubs, 78 (58%) at community organizations, 77 (55%) at coffee shops and restaurants, 71 (51%) at professional networks, 64 (46%) at the gym, 49 (35%) at church, and 30 (21%) at bathhouses.

Regarding sexual behaviors and risk reduction strategies, 100 of 140 men (71%) reported being single at baseline. The median number of male sex partners in the past 6 months was 2, with a mean of 6 and a mode of 1 (SD=14.064). Of 59 men who reported having insertive anal sex with their last male partner, 25 (42%) said they used a condom the whole time, whereas 35 (58%) of the 60 men who reported having receptive anal sex with their last male partner said they used a condom the whole time. Moreover, 89 of the 125 (71%) men reporting a male sex partner in the past 6 months said that they discussed HIV status with their last male sexual partner. Respondents reported an average of 4.1 on a 1-5 Likert scale (4=agree and 5=strongly agree) for both condom use with different status partners and condom use with unknown status partners. Regarding intentions to use risk reduction strategies other than condoms in the past 6 months, on a 1-5 Likert scale, baseline respondents reported a 3.5 average on serosorting (3=neutral and 4=agree), 2.6 on negotiated safety agreements (2=somewhat disagree and 3=neutral), and 2.5 pulling out when topping. Only 3 men stated they were on PrEP at baseline.

We found statistically significant differences (*P*<.05) between the intervention and control conditions at baseline for 3 variables theorized to be linked to sexual behavior outcomes—age, HIV status, and having someone to talk about dating and relationships. These 3 variables were included as covariates in the linear regression and logistic regression analyses.

### Intervention Effects

Intentions and Disclosure

In comparision with the control group condition, Real Talk participants were more likely to disagree that they had intended in the last 6 months to bottom without a condom with a partner of unknown status (mean difference=−0.608, 95% CI=−1.23 to −0.09, *F*_1,94_=5.4, *P*=.02), have their partner pull out when bottoming with a partner of unknown HIV status (mean difference=−0.651, 95% CI=−1.25 to −0.05, *F*_1,86_=4.64, *P=*.03), and pull out when topping a partner of unknown status (mean difference=−0.644, 95% CI=1.2 to −0.08, *F*_1,88_=5.23, *P*=.03), and have anal sex without a condom with a positive man who was on HIV medications approached significance (mean difference=−0.471, 95% CI=−0.95 to 0.01, *F*_1,99_=3.77, *P*=.055; see Multimedia Appendix 5). Real Talk participants were also significantly more likely to disagree with the following statement: “I will sometimes lie about my HIV status with people I am going to have sex with” (mean difference=−0.411, 95% CI=−0.79 to −0.03, 4, *F*_1,100_=1.54, *P*=.04). There were no other significant differences on the remaining 11 disclosure Likert scale questions, disclosure of HIV status with the last male sex partner, or the partner communication self-efficacy scale.

### Condom Use and Other Risk Reduction Practice

There were no significant differences between Real Talk and control participants regarding condom use for insertive or receptive anal sex at last sexual encounter and the number of partners in the past 6 months with whom they always used condoms for insertive and receptive anal sex. Nor were there any signficant differences regarding use of non-condom-based risk reduction practices, although there were insufficient data to support detailed analyses of the number of times respondents used risk reduction strategies by partner HIV status and type.

### Mediators

Men in the control condition were significantly more likely to agree that they had less concern about becoming HIV positive because of the availability of antiretroviral medications (mean difference=0.778, 95% CI=−1.47 to −0.08, *F*_1,110_=4.84) *P*=.03), post-exposure prophylaxis (mean difference=0.826, 95% CI=−1.33 to −.33, *F*_1,108_=10.76, *P*=.001), and PrEP (mean difference=0.658, 95% CI=−1.31 to −.01, *F*_1,106_=4.06, *P*=.05). Control condition participants were also significantly more likely to agree with the statement “sheepskin condoms are better than latex condoms in preventing HIV transmission” (17.6% vs 4.8%, exp (b)=7.10, *P*=.03). There were no significant differences between the intervention and control conditions on stress, self-esteem, negative and positive coping, self-efficacy, or social support variables.

### User Satisfaction

Real Talk participants provided higher satisfaction ratings on a 1 to 5 scale than control condition participants in the 4 principal user experience categories: enjoyment (4.25 vs 3.31, *t*_200_=7.02, *P*<.001), presentation (4.30 vs 3.58, *t*_190_=5.53, *P*<.001), held attention (4.07 vs 3.40, *t*_190_=4.43, *P*<.001), and clarity (4.39 vs 3.68, *t*_200_=5.68, *P*<.001). Real Talk participants also gave significant higher ratings on a 1 to 7 scale on design (6.10 vs 4.68, *t*_166_=6.23, *P*<.001), ease of use (6.07 vs 5.44, *t*_196_=2.09, *P*=.003), usefulness (6.36 vs 5.37, *t*_177_=4.65, *P*<.001), potential to help people lower their sexual health risks (6.39 vs 5.30, *t*_170_=5.28, *P*<.001), and learning something new (83.4% vs 64.2%, χ^2^_4_=22.8, *P*<.001).

## Discussion

### Main Findings

Our study demonstrates that a technology-delivered sexual health promotion program (Real Talk) resonates with black gay men/MSM and supports self-reflection on sexual health and relationship issues. The data further support our first 2 study hypotheses, with Real Talk participants demonstrating, in comparison with the control condition, less intention to have risky forms of anal sex with unknown status partners and to lie about their status to their partners. These results suggest that despite a well-documented trend toward decreased concern about HIV infection and increased sexual risk behaviors in MSM communities [17,29-32], it is possible to reengage MSM on HIV prevention issues. This finding is of particular importance given the results of 2 recent studies that describe how black MSM have higher levels of treatment optimism than men of other race/ethnicities and link these beliefs to increased sexual risks [33,34].

Nonetheless, we found no significant differences between Real Talk and control participants regarding actual risk reduction practices in the past 6 months. There are several possible explanations as to why Real Talk did not generate lower sexual risks. It may be that a one-time, 2-hour intervention is insufficient to support behavioral change on its own, and that Real Talk might contribute more effectively to improved sexual behavioral outcomes if combined with other strategies, including treatment as prevention modalities [35-37]. A second interpretation of the lack of sexual behavioral change may relate to self-presentation and social desirability [38]. Our outcome study data suggest that Real Talk’s extended discussion of the benefits and risks of different sexual harm reduction strategies (eg, serosorting, strategic positioning, and pulling out before ejaculation), including the relative risk ranking of these activities, results in heightened awareness of the risks associated with these acts. This realization may make men less willing to admit that they intend to engage in these practices, a possibility exacerbated when reporting such findings in a survey conducted at an HIV prevention organization. It is also interesting to note that despite its normatively neutral presentation of risk reduction strategies other than condoms, Real Talk did not cause participants to increase their utilization of these strategies, much as participating in needle exchange programs does not lead to increased risk or harm, despite public perceptions to the contrary [39].

### Limitations

This study has several limitations. One concern is the sample size. Despite monetary compensation and extensive recruitment and follow-up activities, we did not achieve our target sample size of 240, and our retention rate of 62% was substantially less than the 80% to 90% retention rates we have achieved in similar outcome studies of eHealth interventions in communities of color over the past 5 years. In addition, 20% of men reported having had no male sex partner partners in the past 6 months, and not all men had sex with positive, negative, and unknown status partner or practiced both receptive and insertive anal sex. As a result, our sample does not support detailed analyses of the number of times respondents utilized different risk reduction strategies according to partner HIV status, differences in risk reduction strategies based on relationship status and particular sexual acts (eg, insertive vs receptive anal sex), and the reasons men did not use condoms with their most recent sexual partners. It is possible that with greater power, we would have been able to detect more nuanced differences in sexual risk behaviors between the Real Talk and control groups.

A second concern is the study’s reliance on self-reported data for its outcomes measures—even with computer-administered instruments, such data may not always accurately capture respondents’ actual sexual behavior and may include inconsistent responses [40]. For example, in our study’s post survey, 88 men reported having more than one male sexual partner in the past 6 months in the opening question of the sexual history section. Yet, in the concluding sections on the details of their last 3 sexual partners, only 50 men reported having had a second male partner. This difference could be a result of recollection, but perhaps is more likely a reflection of survey burnout—after 30-45 minutes, men may have preferred to respond that they had not had a second or third partner in the past 6 months to get to the end of the survey more quickly.

A third limitation is our focus on sexual behavior outcomes. We designed our study in this manner to obtain evidence to support Real Talk’s inclusion in CDC’s DEBI library. However, a growing literature demonstrates that black MSM, in comparison with MSM of other race/ethnicities, have higher rates of HIV infection due to structural factors (eg, health care access, culturally component care, and HIV/STI testing rates) rather than higher levels of sexual risk [41,42]. It is possible that Real Talk may support men’s ability to negotiate such structural barriers to sexual health, but we were unable to address these dimensions in our study.

A fourth limitation relates to Real Talk’s technological specifications. Our decision to develop a relatively long and unidirectional program (ie, each module’s content directly builds on that of preceding modules), rather than a shorter and more flexible app for mobile phones, was based on the promising preliminary efficacy findings of the development team’s similar, 2-hour computer-delivered versions of the SiSTA/SiHLE/WiLLOW trilogy [10-12]. At the same time, our development team believed that user experience optimization for a 2-hour long program with extensive video content and interactive components required desktop or tablet platforms rather than a mobile phone app. The lack of a mobile phone app version of Real Talk may have limited men’s willingness to participate in the study and may also limit the ability of practitioners and black MSM to access the program in the future.

### Conclusions

In recent years, researchers and policy makers have called for the expansion of culturally appropriate HIV-related programs, social marketing campaigns, and health care services to address the elevated HIV rates in black MSM communities [5,43]. This study demonstrates that holistic, harm-reduction-based, eHealth interventions can reengage men on HIV prevention issues in the current age of treatment optimism, safer sex burnout, and multiple risk reduction strategies. This possibility of reaching black MSM through a technologically contemporary intervention is critical given that many men meet their sexual partners using the Internet and mobile phone apps [44-46] and increasingly rely on technology for their health information seeking [47,48].

Seeking to promote program utilization and longevity, we designed Real Talk to be a flexible tool focused on skills acquisition, self-reflection, and participant-generated content that enables men to address emerging sexual health issues without requiring a major reworking of the intervention. Real Talk’s modular format also facilitates its utilization in conjunction with a broader array of prevention activities, including HIV testing and counseling, online outreach, and community-level programs, and may help address the currently low levels of PrEP utilization in black MSM communities [17]. In our future research, we are particularly interested in examining the processes through which interventions produce changes, including, in the case of Real Talk, the possible connection between sexual health and experiences with stigma and intersectionalities.

Our interest in identifying mediating variables and key intervention components [49,50] stems from the realities of real-world program implementation, where providers often combine pieces of evidence-based programming to complement their existing programs and services rather than implementing a complete evidence-based intervention. We observed this dynamic during our outcome study site trainings, where all 4 organizations expressed a desire to integrate parts of both the face-to-face curriculum and the computer/tablet-delivered programs into their everyday activities upon the completion of the study. By examining the connections between outcomes and real-world utilization patterns, including hybrid formats that combine face-to-face and technology-delivered activities, researchers may be able to identify what might be called health promotion chunks (on cognitive chunking more generally, see [51]) that support healthy practices and outcomes in diverse utilization contexts and populations. The identification of efficacious health promotion chunks would help inform the development of an emerging wave of multidirectional, mobile sexual health apps for MSM [52-54]. Such products would not only help support the scaling up of cost-effective HIV prevention/sexual health promotion in resource-tight environments, but could provide a model for health promotion programs more generally in the Internet age.

## Appendix

Multimedia Appendix 1 Real Talk content.

Multimedia Appendix 2 Real Talk screenshot 1.

Multimedia Appendix 3 Real Talk screenshot 2.

Multimedia Appendix 4 Real Talk outcome study flowchart.

Multimedia Appendix 5 Real Talk outcome study findings.

## Abbreviations

ART: antiretroviral therapy
CDC: Centers for Disease Control and Prevention
DEBI: Diffusion of Effective Behavioral Interventions
GHB: gamma-hydroxybutyric acid
MSM: men who have sex with men
PrEP: pre-exposure prophylaxis
SiHLE: Sisters Informing Healing Living and Empowering
SISTA: Sisters Informing Sisters about Topics on AIDS
STI: sexually transmitted infections
WiLLOW: Women Involved in Life Learning from Other Women
