# Supplementary material for: Preliminary Findings of a Technology-Delivered Sexual Health Promotion Program for Black Men Who Have Sex With Men: Quasi-Experimental Outcome Study
Source: JMIR Public Health Surveill. 2017 Oct 24;3(4):e78. doi: 10.2196/publichealth.7933 (PMC5676034; doi:10.2196/publichealth.7933)
Supplement: Multimedia Appendix 1 [file publichealth_v3i4e78_app1.pdf]

Multimedia Appendix 1

Real Talk Components

Module	Content (2 hours)
1. My Community	15 minutes: Welcome-From Self-Awareness to Self-Actualization-Pitfalls (presentations), Social Roles & Identities (click-drag activity), Role Conflicts (video), Personal Values (listing activity), Community Assets and Resources (presentation, video & rating activity), My Community (health resources rating activity), Stigma & Barriers (group video), Conclusion-Know Your Community (video)
2. Achieving Our Goals	15 minutes: Goals (presentation), Envision You (breathing/reflection exercise), Imagine Yourself (listing activity), Goals & Values (click-drag activity), What is Advocacy-Advocacy Action Plan (presentations), Make Your Own Action Plan (3-step listing/rating activity), Conclusion-Finding Resources (video)
3. Stress & Social Support	20 minutes: What is Stress-Reacting to Stress-Coping with Stress (presentations), Reacting to Stress (click-drag activity), RELAX & DECIDE Models (presentations/videos), Your Social Support Network (presentation), Goals & Social Networks (click-drag and listing activity), Creating a Social Support Action Plan (group video/reflection), Conclusion-Coping with Stress (video)
4. Sexual Harm Reduction	40 minutes: Having a Healthy Sex Life (reflection), How We Make Decisions (presentations), Discussing Sex (video), Good Things About Sex (presentation), STIs (interactive presentation), 6-step Harm Reduction (presentation), Why Do You Have Sex (reflection/listing activity), Applying 6-Step Harm Reduction to Anal Sex (presentation/click-drag activity), Condom Lab (videos), Conclusion-Know Your Status, Findr (online hook-up role play game)
5. Communication	15 minutes: Communication (presentation), Communication Styles (videos), Get the Conversation Going (presentation), HiLo (sexual communication game), Conversation & Sex (presentation), Conclusion-Sexual Communication
6. Building Healthy Relationships	15 minutes: Building Healthy Relationships (presentations), Real Talk session on relationships (video), All That or Wack: Healthy v. Unhealthy Relationships (game/presentation), Is This a Healthy Relationship (video), Relationship Coaching (click activity/group video), Build Your Guy & Relationship (click-drag activity), Conclusion-Sexual Health & Your Life Goals
