# Supplementary figures and images for: Preliminary Findings of a Technology-Delivered Sexual Health Promotion Program for Black Men Who Have Sex With Men: Quasi-Experimental Outcome Study
Source: JMIR Public Health Surveill. 2017 Oct 24;3(4):e78. doi: 10.2196/publichealth.7933 (PMC5676034; doi:10.2196/publichealth.7933)

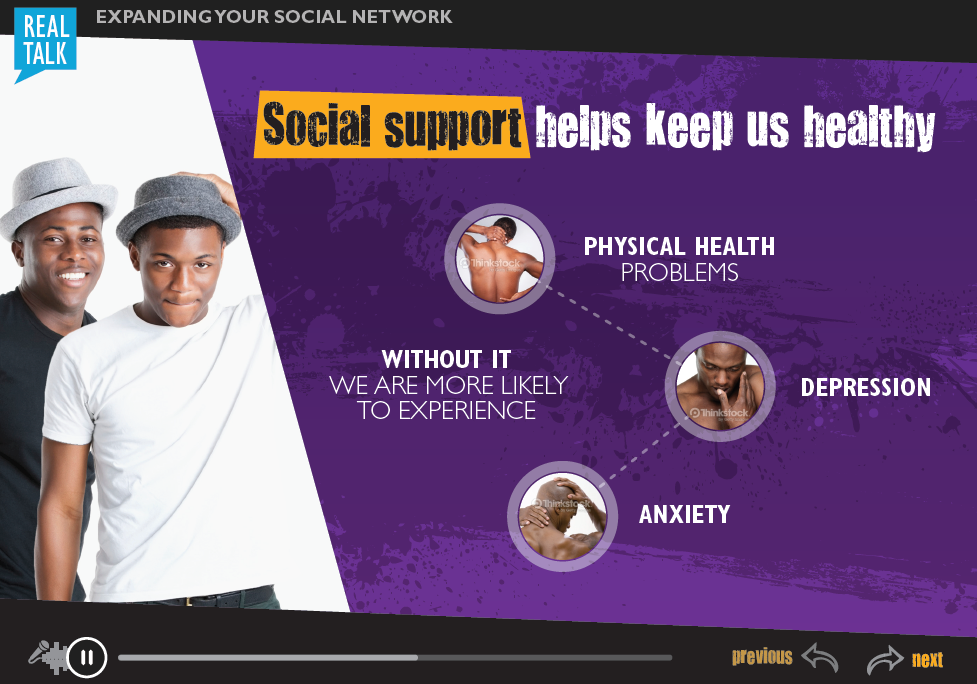

Supplement: Multimedia Appendix 2 [file publichealth_v3i4e78_app2.PNG]

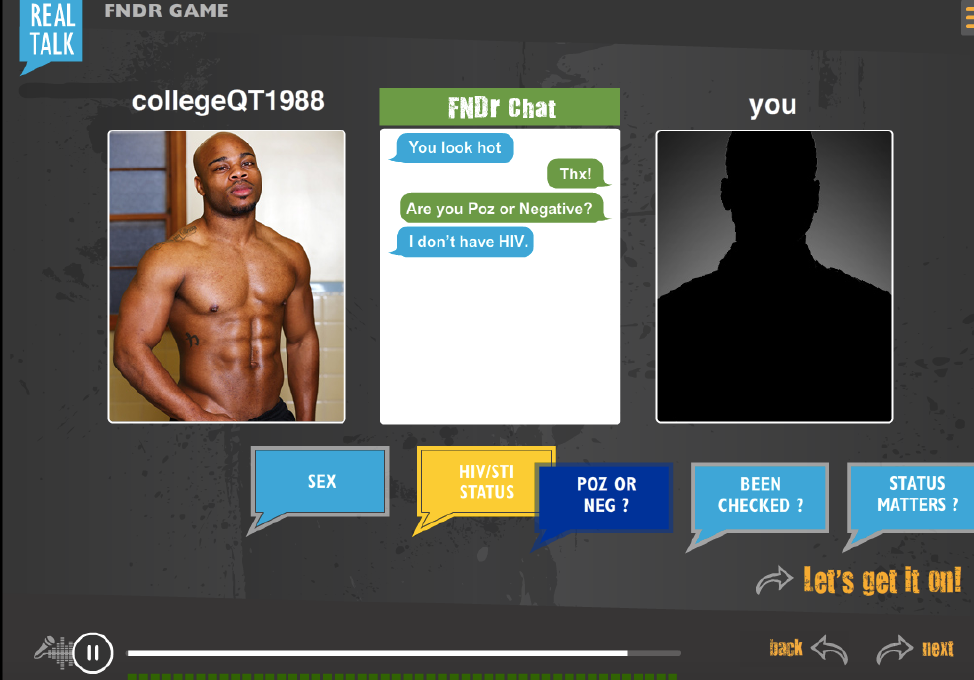

Supplement: Multimedia Appendix 3 [file publichealth_v3i4e78_app3.PNG]

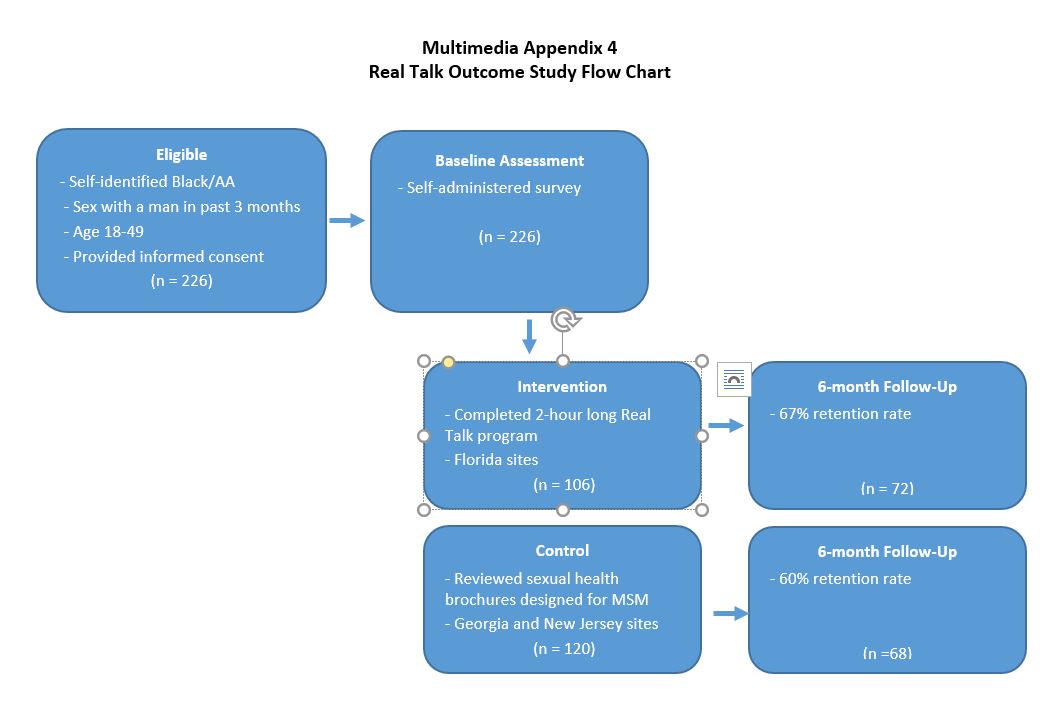

Supplement: Multimedia Appendix 4 [file publichealth_v3i4e78_app4.JPG]
