# Supplementary material for: Preliminary Findings of a Technology-Delivered Sexual Health Promotion Program for Black Men Who Have Sex With Men: Quasi-Experimental Outcome Study
Source: JMIR Public Health Surveill. 2017 Oct 24;3(4):e78. doi: 10.2196/publichealth.7933 (PMC5676034; doi:10.2196/publichealth.7933)
Supplement: Multimedia Appendix 5 [file publichealth_v3i4e78_app5.pdf]

Multimedia Appendix 5
Outcome Study Findings

Variable	Unadjusted mean (SD)/Percentages		Adjusted[a] Mean Difference (D) (Linear Regressions)	% Relative Change (95% CI)[b] (Linear Regressions)	Adjusted Odds (95% CI)[c] (Logistic Regressions)	Test statistic [d]	*P*
	Real Talk (I)	Control (C)					
**Intentions, Past Six Months**	.						
Likert scale affirmations (1 =strongly disagree, 5 = strongly agree)							
Unknown status partner: bottom without a condom	1.72 (1.22)	2.16 (1.30)	-.608 (-1.23, -.09)	-28.15 (-56.28, -4.26)	NA	5.402	**.022**
Unknown status partner: bottom without condom if partner withdraws before ejaculation	1.76 (1.25)	2.29 (1.41)	-.651 (-1.25, -.05)	-28.43 (-53.04, -2.33)	NA	4.637	**.034**
Unknown status partner: top without condom if withdraws before ejaculation	1.89 (1.27)	2.51 (1.47)	-.644 (-1.2, -.08)	-25.66 (-46.61, -3.27)	NA	5.233	**.025**
Positive partner: Anal sex without condom if partner on HIV medications	1.85 (1.31)	2.35 (1.40)	-.471 (-.95, .01)	-20.04 (-39.47, 0.43)	NA	3.767	.055
**Last Sexual Partner**							
Used condom for insertive anal intercourse	43.2	50.0	NA	NA	.720 (.27, 1.91)	.720	.510
Used condom for receptive anal	56.25	62.25	NA	NA	.950 (.34, 2.64)	.950	.992

Intercourse							
**Disclosure**							
Willingness to lie about HIV status	1.50 (.89)	1.72 (1.03)	-.411 (-.79, -.03)	-23.90 (-45.16, -1.77)	NA	4.536	**.036**
Partner communication self-efficacy scale	19.98 (5.73)	20.16 (5.33)	.216 (-1.76, 2.19)	1.07 (-6.97, 13.05)	NA	.047	.980
Discussed HIV status with last male partner	74.00	75.86	NA	NA	.950 (.40, 2.26)	.950	.908
**Knowledge**							
Sheep skin condoms better than latex condoms in preventing HIV transmission	4.76	17.65			7.10 (1.21, 41.84)	7.10	**.027**
**HIV Concern**							
Less concerned about HIV infection because of medications	2.22 (1.85)	2.58 (1.76)	-.773 (-1.47, -.08)	-29.96 (-55.41, -3.18)	NA	4.841	**.030**
Less concerned about HIV infection because of PEP	1.84 (1.07)	2.40 (1.64)	-.826 (-1.33, -.33)	-34.42 (-54.09, -14.08)	NA	10.76	**.001**
Less concerned about HIV infection because of PREP	2.02 (1.32)	2.50 (1.94)	-.658 (-1.31, -.01)	-26.32 (-51.09, -0.41)	NA	4.061	**.046**

a Adjusted by covariates: corresponding baseline variable, age, HIV status, and having someone to talk to about dating and relationships.
b % Relative change (RC) = [D/C * 100 %] and 95 % Confidence Interval around the % relative change
c Adjusted odds ratios calculated with the control comparison condition as the referent (OR = 1.0)
d Test statistics listed consist of F for continuous variables and Exp(B) for count and categorical variables
